# A Vaginal Inlay for Reduction of Stress Urinary Incontinence: Outcome of a Randomized Clinical Trial and Four User Acceptance Studies

**DOI:** 10.1155/2021/8822186

**Published:** 2021-02-15

**Authors:** Aino Fianu Jonasson, Karin Bryder, Elisabeth Sthengel

**Affiliations:** ^1^Division of Obstetrics and Gynaecology, Karolinska University Hospital, Huddinge, 141 86, Stockholm, Sweden; ^2^Invent Medic Sweden AB, Medicon Village, 223 81, Lund, Sweden

## Abstract

A multicenter, randomized, controlled clinical trial and four postmarket user acceptance investigations were carried out to document the safety, performance, and user acceptance of Efemia Bladder Support, a novel vaginal inlay for the temporary reduction of stress urinary incontinence (SUI). The clinical investigation enrolled 97 women diagnosed with SUI, randomized 3 : 1 to either treatment or standard care (control). The primary endpoint was reduction of urine leakage, measured as change in pad weight baseline week compared with treatment week. Secondary endpoints were treatment success, calculated as the percentage of subjects with >70% reduction in pad weight, reduction in incontinence episodes, and quality of life (QoL). 75 women (77%) completed the clinical investigation. No serious adverse events occurred. The treatment group reached a 55% (*p* < 0.001) mean reduction of total leakage compared to the control arm. A subanalysis, involving only leakage during provocation testing (coughing and jumping), showed a 67% (*p* < 0.001) mean reduction of leakage. No significant effect on QoL could be observed. 51% of the women answered “yes” to the question if they would use the device to reduce SUI. The user acceptance of the device was further investigated in four postmarket studies, using an improved device design with a slimmer centerpiece and a thinner handle, while keeping the effect achieving parts of the device unchanged. An average of 74% of the 102 participants in the postmarket studies reported that they were likely to continue using Efemia. The highest user satisfaction was seen in the two studies evaluating the use of Efemia during exercise, where 83% and 88% of the women were likely to continue using Efemia. It can be concluded that Efemia is a safe, well-tolerated, and effective alternative for reducing SUI, both in everyday life and during physical exercise.

## 1. Introduction

Urinary incontinence (UI) in women is a distressing condition that restricts quality of life and interferes with daily activities. In a review of reported UI prevalence in studies, Milsom et al. found that most studies reported a prevalence of female UI in the range of 25 to 45%. Stress urinary incontinence accounted for approximately half of the UI cases and the prevalence increases with increasing age [1]. However, SUI occurs also in younger, physically active women. According to Abrams et al., it is clear that high impact exercise leads to stress UI, with a dose-dependent deleterious effect, while low-impact sports might be protective [2]. In a review of 36 publications addressing the associations between physical activity and SUI, Chisholm et al. found evidence for increased rates of SUI among women who are physically active [3]. In a study quantifying SUI in 104 young female athletes, 52% of the participants self-reported SUI according to the ICQ_UI_SF score [4]. These results show the importance of not only evaluating device performance on the target group of elderly women clinically diagnosed with SUI but also including younger, active women that experience urine leakage during exercise, but not necessarily define themselves as being incontinent.

There are several treatment options for SUI available. In clinical practice, it is the convention that nonsurgical therapies are tried first because they usually carry the least risk of harm. Containment devices (absorbent pads, urinary catheters, and intravaginal devices) play an important role, especially for individuals who prefer to avoid the risks of interventional treatments, or in whom active treatment is impossible for any reason. [5]. Insertion of a synthetic sling to give support to the midurethra is currently the first in line recommended surgical approach [5, 6]. However, as more women are treated, concerns of the safety of the surgery have been raised. In a data analysis of incontinence surgery registered in The Swedish National Registry for Gynecologic Surgery (GynOP), presented at the ICS 2019 conference, S. Zacharias et al. reported that, out of 4,160 Swedish women that went through incontinence surgery during 2017, 681 women (16%) responded that they either had a complication, were worse off in their incontinence than before, or were unhappy with the results. The most common problems were urinating problems, pain, infection, and rupture/erosion of the tape in the vagina [7]. It is clear from this data that there is a need for effective nonsurgical alternatives for treatment or alleviation of SUI.

In a review of studies on the use of vaginal pessaries for the management of SUI, Ghadeer et al. concluded that they are an effective alternative to surgery if they are fit properly [8]. Several models of vaginal inserts designed to give support to the urethra or bladder neck to temporarily reduce urine leakage have been evaluated in clinical studies. Examples are Uresta [9], Diveen [10], Contrelle [11], Impressa [12], and Contiform [13]. Due to differences in vaginal anatomy and personal preferences, it cannot be expected that one design will suit all women. The introduction of Efemia gives women a new choice of device, with a handle for easy self-positioning, ensuring correct placement at the midurethra.

The aim of the present clinical investigation and postmarket studies was to evaluate the safety, efficacy, and usabillity of Efemia.

## 2. Materials and Methods

### 2.1. Investigational Device

**Figure 1:**
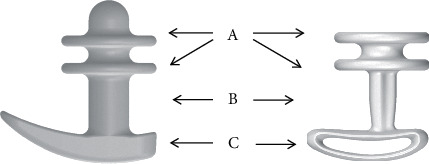
Investigational device, size 30 mm. Left: the design used in the clinical investigation; right: the design used in the postmarket studies. A = support rings, B = midsection, and C = handle.

Efemia Bladder Support is intended to temporarily reduce SUI in women by supporting the midurethra through the vaginal wall. It is reusable and can be used continuously up to 16 hours a day or on occasion when urine leakage is expected. It is made from medical grade silicone rubber and is available with three support ring diameters: 30 mm, 35 mm, and 40 mm. A first design was used during the clinical investigation TVS1000. After the clinical investigation, the device design was improved with a slimmer centerpiece and a thinner handle, while keeping the effect achieving parts of the original trial device unchanged. The revised device was used in the postmarket studies TVS2000, TVS3000, TVS4000, and TVS5000.  Figure 1 shows the two device designs. The two rings (A) support the midurethra through the vaginal wall, thereby preventing involuntary urine leakage upon abdominal pressure. The midsection (B) positions the device correctly at the urethral knee. The handle (C) holds the device in place and is placed against the vaginal introitus. The handle is also used as an aid at insertion and removal of the device.

### 2.2. Study Design of the Clinical Investigation TVS1000

#### 2.2.1. Study Title

The study title is an Open Randomized Controlled Multicenter Clinical Investigation with an Intravaginal Device for Stress Urinary Incontinence in Comparison to Using Standard of Care.

#### 2.2.2. Study Population

All study participants were diagnosed with stress urinary incontinence, had normal voiding, were above 18 years, and leaked at least 10 g per 24 hours. Women with dominated urgency or neurogenic incontinence, hysterectomized, with a pronounced prolapse, pregnant, with a vaginal infection, or with a history of not being able to use tampons were excluded from the study.

#### 2.2.3. Randomization

Subjects fulfilling the exclusion and inclusion criteria were randomly assigned at a 3 : 1 ratio to either the TVS treatment group or the standard-of-care (SoC) group at visit 2, using block design. Each site was provided with closed envelopes with consecutive numbering. Those assessing the outcomes were blinded to treatment allocation until the database was validated and locked.

#### 2.2.4. Primary Endpoint

Absolute reduction in pad weight from the run-in week (week 1) compared with the final week (week 3). The weight of the pad is defined as the mean weight over the entire week of measurement.

#### 2.2.5. Secondary Endpoints

  Overall success rate, defined as at least 70% reduction in pad weight from run-in to the final week (week 3)
  Reduction of SUI episodes
  Reduced incontinence impact on QoL, measured using the validated patient reported outcome questionnaires EQ-5D-5 L, IIQ-7, and UDI-6

#### 2.2.6. Study Size and Statistical Analysis

The aim was to enroll 96 female study subjects. A study size of 96 patients was calculated using a standard deviation of 9 g, a power of 90%, a significance level of 5%, and a 20% dropout rate. A mean leakage reduction of 13 g was assumed for the active group and 5 g for the control group (MIREDIF = 8 g). The full analysis set, defined as randomized subjects with at least one test measurement after randomization (FAS), was used for safety primary and secondary efficacy endpoints. A subanalysis, involving only leakage during provocation testing, was also planned. Center and treatment were used as covariates in the analysis of the primary variable. Hypothesis was tested using a two-sided Student's *t*-test with a 5% significance level. No adjustments for multiplicity were performed. Outliers were not excluded. Data from prematurely withdrawn subjects was included in the analysis as far as possible. No imputation of missing data was performed.

#### 2.2.7. Study Procedures

The clinical investigation was conducted at four clinics with Aino Fianu Jonasson, Dr, Md, PhD, urogynecology specialist at Karolinska University Hospital, as coordinating investigator. Participants were recruited via advertisements in newspapers and on Facebook. At the screening visit, a medical and surgical history, a physical examination (including gynecological examination), and a confirmation of the diagnosis of SUI were performed, followed by a confirmation of the inclusion/exclusion criteria. SUI was diagnosed by pad testing and measurement of residual urine after filling the bladder with 300 ml saline and provoking urine leakage by coughing, jumping, and rinsing hands in cold running water. A positive cough/jump test indicates stress incontinence while a positive rinsing test indicates urge incontinence. Calibrated scales were distributed to the participants for weighing pads. Baseline data was collected during the first week. During week 2, the women in the TVS group selected the device size and practiced how to use the device prior to the start of treatment at week 3. The control group continued with conventional treatment, i.e., using pads during weeks 2 and 3. For women who were menstruating, the study was interrupted, due to the fact that menstruation could affect the weight of the pads and consequently the study data. Specific instructions for re-entering the study were given to the subjects prior to start. The women used a diary to record their incontinent episodes, pad weight, physical activities, and general observations during the first three weeks of the study. They also performed a daily provocation test by coughing 10 times and jumping in place (or sit and stand if unable to jump) 20 times with a full bladder. At the weekly visits to the clinic, the women were asked QoL questions (IIQ-7, UDI-6, and EQ-5D-DL) and were asked to rate their experience of the device. After completing week 3, women in the control group were offered to use the device for two weeks. If they chose to do so, they switched over to a SoC-TVS group and went to a 5th visit to the clinic to answer questions regarding their experience. Both the TVS and the control group had a follow-up telephone call after 8 weeks.

#### 2.2.8. Safety Monitoring

Subjects were carefully monitored for the occurrence of adverse events (AE) during the investigation period from randomization to the completion of follow-up. The clinical investigators collected AE information using nonleading questions. Events directly observed or spontaneously volunteered by subjects were also recorded.

#### 2.2.9. Ethical Considerations

The investigation was conducted in compliance with the ethical principles of the latest revision of the Declaration of Helsinki as well as ISO 14155 : 2011. The study was reviewed and permission granted by Ethical Committee and Competent Authority prior to start (EC registration #2016/1899/31/1, CA registration#: CIV-16-10–017304). Clincaltrial.gov identifier: NCT03186651.

### 2.3. Study Design of Postmarket Studies: TVS2000, TVS3000, TVS4000, and TVS5000

The four postmarket studies were designed as observational user satisfaction studies. The identity of the participants was not revealed to the sponsor (Invent Medic Sweden AB). The subject recruitment, informed consent, subject ID-log, and distribution of questionnaires and study products were handled by independent study administrators at the Ladulaas Clinic (TVS2000), Aller Media (TVS3000), Tilling Träning (TVS4000), and the Urotherapy Clinic at Skelleftea Hospital (TVS5000). Participants received a free package containing the three sizes of Efemia. Participants recruited from the Aller Media test panel collected loyalty points that could be used for purchase in the Aller Media webshop. No other compensation or rewards were given for participation.

#### 2.3.1. Sample Size

The sample size calculation in the TVS2000 and TVS3000 study was based on the standard deviations for the average IIQ-7 score in the TVS1000 study. A study size of 25 subjects is required to detect a significant difference of at least 25% with a power of 80% and a significance level of 5%, expecting a standard deviation of 32%.

The sample sizes of the TVS4000 and TVS5000 studies were not based on power calculations, since the primary endpoint was user satisfaction and they do not contain any hypothesis evaluations. It was estimated that a sample size of 20 women would represent a sufficient basis for evaluating user satisfaction during exercise.

#### 2.3.2. Study Objectives

The first two studies, TVS2000 and TVS3000, had the objective to investigate the effect on quality of life (measured as reduction in IIQ-7 score) and user satisfaction when using Efemia in everyday life. The participants answered the IIQ-7 questionnaire before using Efemia and after 4 weeks of usage. The other two studies had the objective to evaluate user satisfaction when using Efemia during physical exercise.  Table 1 shows a comparison of the design of the four studies.

#### 2.3.3. Ethical Considerations

The studies were designed and performed by adhering to the ICC/ESOMAR international code of marketing and social research [14]. In addition, ethical approval was obtained for the TVS2000 study because it included participants from the previous clinical investigation TVS1000 (EC ref: EPN Lund, Dnr: 2018/959).

## 3. Results and Discussion

### 3.1. Results of TVS1000 Clinical Investigation

A total of 191 women at four clinical sites were screened for eligibility. Of these, 97 women met all the inclusion and none of the exclusion criteria and were thereafter randomized.

The most common screening failure was <10 g urine leakage during 24 h (*n* = 25), followed by prolapse reaching hymen when coughing (*n* = 12). Recruitment started in Feb 2017 and the study was completed in Jan 2018. The women were randomized 3 : 1 to either use the device or standard care (SoC).

The groups had similar baseline demographics and clinical characteristics, and the mean age was 54.7 years. The mean leakage weight at the controlled provocation test during the screening visit was 53.5 g. The baseline characteristics and demographics are shown in Table 2.

**Figure 2:**
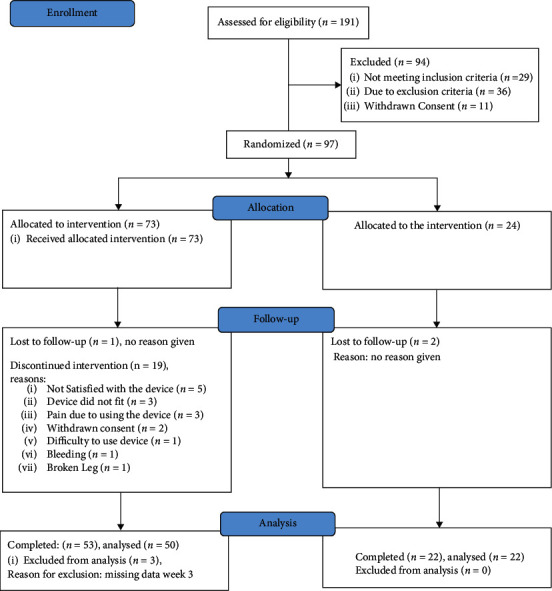
TVS1000 participant flow.

20 subjects in the TVS group and 2 subjects in the control group discontinued the investigation. A chart of participant flow and reasons for discontinuation is shown in Figure 2.

### 3.2. Primary Outcome

The absolute reduction in mean pad weight from baseline (week 1) compared with the final week (week 3) was 18.2 g. The estimated treatment effect compared with the control group for total leakage was 12.4 g (55%) with a *p* value of 0.0005 in favour for the TVS group.

The absolute reduction in pad weight during provocation testing was 28.2 g (67%, *p* < 0.0001). Analysis of covariance for the variables: treatment group, site, age, weight, and residual volume showed no association between the covariate and the primary outcome measure. The outcome is summarised in Table 3.

### 3.3. Secondary Outcome

Overall success rate, defined as at least 70% reduction in pad weight from the run-in week to the final week (week 3): 42% of the subjects in the TVS arm and 4% of the subjects in the control arm had more than 70% reduction in pad weight with a *p* value = 0.0008 in favour for the TVS group.

The number of SUI episodes at week 3 compared to baseline was reduced with a median of 28% (*n* = 52, max-min, -95-150%) for the TVS group and 0% (*n* = 23, max-min, -46-557%) for the control group with a *p* value = 0.0019 in favour for the TVS group.

Incontinence Impact Questionnaire (IIQ-7): IIQ-7 score was reduced with 10% for the TVS group and 12% for the control group. There was no statistical difference between the TVS group and the control group (*p*=0.414).

Urogenital Distress Inventory (UDI-6): UDI-6 score was reduced with 12% for the TVS group and 18% for the control group. No statistical difference between TVS group and control group was observed (*p*=0.1971).

### 3.4. Safety

A total of 92 women were exposed to the device for two weeks during the investigation. No device-related serious adverse effects occurred. 32 of the 92 subjects using the device reported device-related adverse effects (45 episodes). The most frequent adverse device effect was discomfort (30), followed by bleeding (4), vaginal discharge (4), contusion (4), itching (2), and candidiasis (1). All adverse effects were resolved when TVS use was reduced, except for the single incident of candidiasis that needed medical intervention.

### 3.5. Usability

The usability analysis included all 72 women that had been exposed to the device at any time during the investigation and answered the usability questions. 85% of the women rated the device as easy or very easy to insert or remove. 51% answered “yes” to the question if they would use the device to reduce SUI, and 75% of them would recommend the device to a friend. 58% found the device to be comfortable (acceptable or perfectly acceptable), 13% were neutral, and 29% found it to be unacceptable.

## 4. Results of Postmarket Surveillance Studies: TVS2000, TVS3000, TVS4000, and TVS5000

**Figure 3:**
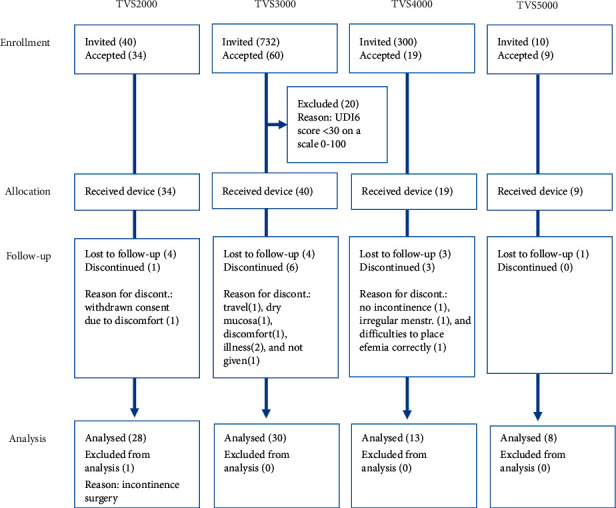
Participant flow, postmarket surveillance studies.

A total of 102 women were enrolled in the four user satisfaction studies. 79 women used Efemia in their daily lives or during exercise and are included in the analysis. All studies were performed during 2019. A chart of participant flow in the four studies is shown in Figure 3.

**Figure 4:**
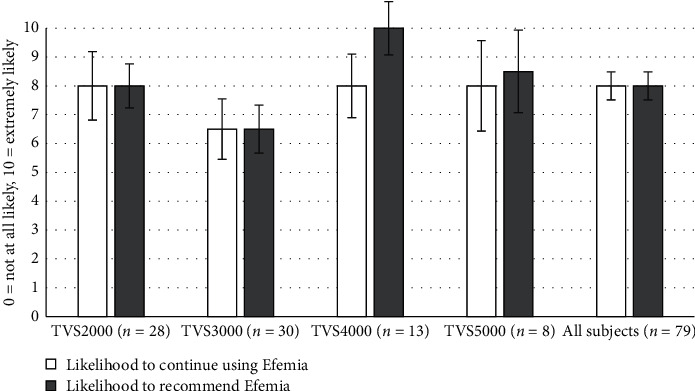
Median gradings of the questions: on a scale of 0–10, how likely are you to continue using Efemia or to recommend Efemia to a friend? Error bars = 95% CI.

User satisfaction was measured as likelihood to continue to use Efemia and to recommend Efemia to a friend. The women were asked to grade their answers 0–10, where 0 = ” not at all likely” and 10 = ” extremely likely.” A grading of 6 or above was regarded as a positive answer. The frequency of women likely to recommend Efemia to a friend in the TVS2000, TVS3000, TVS4000, and TVS5000 studies was 82%, 62%, 92%, and, 100%, respectively. The frequency of women likely to continue using Efemia in the TVS2000, TVS3000, TVS4000, and TVS5000 studies was 68%, 55%, 83%, and 88%, respectively. The median gradings of the likelihood to continue using or to recommend Efemia to a friend, for each study separately and pooled, are shown in Figure 4.

The impact of incontinence on the daily life was measured in TVS2000 and TVS3000 using the validated Incontinence Impact Questionnaire IIQ-7, where the women were asked to grade the impact of their incontinence in 7 areas of everyday life before and after having tried Efemia for 4 weeks. A grading of 0–3 was used, where 0 = not at all, 1 = slightly, 2 = moderately, and 3 = greatly. There was a significant improvement in IIQ-7 scores with a 27% mean decrease in TVS2000 (*p*=0.0015) and a 29% mean decrease in TVS3000 (*p*=0.012).

**Figure 5:**
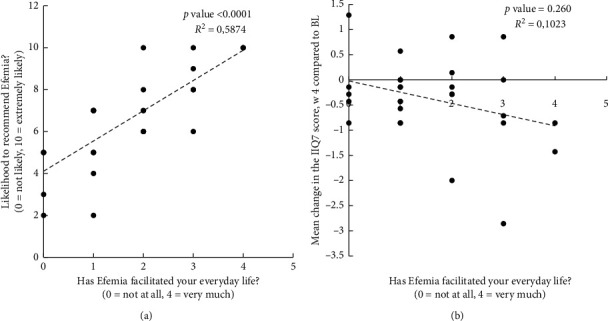
Likelihood to recommend (graph (a)) and mean change in the IIQ-7 score (graph (b)), plotted against facilitation of everyday life, TVS3000 (*n* = 30).

In the TVS3000 study, the effect of Efemia on quality of life was also measured by asking if Efemia had facilitated daily life. A comparison of change in IIQ-7 score with facilitation of daily life showed no significant correlation, while there was a strong positive correlation between facilitation of everyday life and willingness to continue to use or recommend the device with a *R*^2^ of 0.499 and 0.587, respectively. Graphs plotting facilitation of everyday life with change in the IIQ-7 score or likelihood to recommend Efemia are shown in Figure 5.

## 5. Discussion

The clinical investigation TVS1000 confirmed that Efemia Bladder Support is safe and achieves its primary performance objective to reduce involuntary urine leakage with a 55% (*p* < 0.0001) mean reduction of leakage compared to the control group. The subanalysis of leakage during the daily provocation tests showed a 67% (*p* < 0.0001) mean reduction of leakage.

The secondary endpoints for the study were also met. 42% of the subjects in the TVS arm had >70% reduction in pad weight and the number of SUI episodes was reduced by 28%.

The quality of life endpoints in TVS1000 were not met. No clinically significant decrease in the IIQ-7 score, compared to controls, could be detected after 2-week use of the device, while there was a modest but significant decrease of the IIQ-7 score in the two user satisfaction studies TVS2000 and TVS3000, with 27% and 29% reduction, respectively. The published validation of the Swedish form of the IIQ-7 questionnaire reported a strong-to-moderate correlation with treatment satisfaction and reduction of the IIQ-7 score [15]. It was therefore surprising to note that there was no significant correlation between any of the treatment satisfaction variables (willingness to continue using or recommending Efemia), and reduction of IIQ-7 scores in the present investigations. In fact, 6 of the 8 women in the TVS3000 study and 5 of the 7 women in the TVS2000 study, with an increase or no change in the IIQ-7 score after treatment, reported that they were likely to continue using Efemia. It is difficult to understand why anyone will want to continue to use Efemia if it has no effect or even worsens the impact of incontinence on their daily life, as measured with IIQ-7. An explanation might be that women with mild SUI cannot relate to the IIQ-7 questions because most of them are likely to use protective pads in situations where they expect to leak. Therefore, their incontinence has very little impact on their ability to perform daily activities. As one of the women comments, “I think the IIQ-7 questions are wrongly designed. Incontinence is unpleasant but it does not affect my ability to do things.” The inadequacy of IIQ-7 for quantifying the objective severity of SUI is confirmed in the publication by Franco et al. where they found no correlation between reduction in urine leakage (1-hour pad weight) and change in IIQ-7 score [16]. In future studies, the validated ICQ-SF questionnaire might be better suited for assessing incontinence impact on the quality of life in relation to the use of Efemia. However, it can be clearly concluded that the treatment satisfaction was high and that Efemia facilitated the daily lives of the study participants.

No serious adverse device effects occurred. The most commonly reported adverse device effect in the TVS1000 investigation was discomfort (30 reports). Since the TVS1000 investigation, there has been a design change resulting in a lighter and more appealing product. It is therefore interesting to compare the device comfort in the TVS1000 investigation where the old design was used with the TVS2000 study in which the current design was used. In the TVS1000 investigation, 33% of the women considered the device to be uncomfortable while discomfort was experienced by only 7% of the women in the TVS2000 study, where the current device design was used.

The postmarket studies, using the current version of the device, showed a high user satisfaction. This was particularly noteworthy in the two studies where Efemia was used during exercise, where >80% of the women were likely to continue to use Efemia and >90% were likely to recommend Efemia to a friend. However, it is important to note that the evaluation of Efemia during exercise is based on a total of 21 women, using Efemia during cross-fit training and weightlifting. Studies, involving other sports and more women, would therefore be useful for evaluating the use of Efemia by physically active women.

For women diagnosed with SUI, Efemia can be an effective and safe alternative, either temporarily, while waiting for surgery, or to be able to avoid surgery altogether. Furthermore, since Efemia is available “over-the-counter,” it might improve the daily lives of active women experiencing urine leakage during physical exercise, who might not see themselves as generally incontinent and therefore do not seek medical help. A weakness of the TVS3000 investigation is that the device usage was surprisingly low, such that most of the responders had used Efemia only a few times during the 4-week trial period. The reason for this is not clear. It could be either that the participants had very mild incontinence and only experienced urine leakage a few times during the 4-week trial period or that they were not fully dedicated to the study, but rather participated to collect loyalty points from Aller Media.

## 6. Conclusion

It can be concluded that Efemia is a safe, well-tolerated, and effective alternative for reducing SUI, both in everyday life and during physical exercise. It is clear from the studies that even though most women found Efemia comfortable, it does not suit all. Further studies, evaluating comfort and user satisfaction on a larger group of women, over a longer time, could be useful as a guidance in further device development.

## Figures and Tables

**Table 1:** Comparison of study design of the four postmarket surveillance studies.

	Study			
Parameter	TVS2000	TVS3000	TVS4000	TVS5000
Recruitment	Previous participants in TVS1000	Through the Aller Media test panel	Visitors at the gym Tilling Träning	Urotherapist at Skelefteå Hospital
Study size	34	40	19	9
Treatment length	4 weeks	4 weeks	6 weeks	6 weeks
Target group	Women clinically diagnosed with SUI	Women self-diagnosed with SUI	Women with urine leakage during weightlifting	Women with urine leakage during cross-fit training
Study objectives	Change in the IIQ-7 score after 4 w device usage	Change in the IIQ-7 score after 4 w device usage	User satisfaction when using Efemia during weight training	User satisfaction when using Efemia during cross-fit training

**Table 2:** Demographics.

Variable	Treatment arm		
	TVS	SoC	Total
	Mean (SD)	Mean (SD)	Mean (SD)
Number of women	73	24	97
Age (years)	54.8 (10.8)	54.6 (9.38)	54.7 (10.4)
Weight (kg)	78.6 (15.8)	78.2 (17.7)	78.5 (16.2)
Height (cm)	166 (6.22)	167 (6.55)	166 (6.28)
Residual urine (ml)	27.1 (23.9)	29.1 (21.6)	27.6 (23.3)
Urine leakage, controlled provocation test (g)	56.6 (63.3)	43.9 (49.9)	53.5 (60.3)

**Table 3:** Efemia effect on mean urine leakage (average daily pad weight), analysed on the full analysis set.

	SoC (*n* = 22)			TVS (*n* = 50)			Treatment effect
Variable	Week 1	Week 3	Change (g)	Week 1	Week 3	Change (g)	TVS-SoC
Mean total leakage (g)	27.1	22.5	−5.2	27.3	10.5	−18.2	55%
(SD, *p* value)	(33.2)	(20.8)	(34.4)	(33.6)	(21.9)	(35.5)	*p*=0.0005
Mean provoked leakage	32.4	31.2	−1.2	38.2	10.0	−28.2	67%
(SD, *p* value)	(33.0)	(29.2)	(9.5)	(42.6)	(22.1)	(30.0)	*p* < 0.0001

## Data Availability

The primary author and coordinating investigator, Aino Fianu Jonasson, has full access to all data in the study. The data supporting the findings are quality controlled and stored in a locked database. Individual data listings are available upon request.
